# Targeting GATA transcription factors – a novel strategy for anti-aging interventions?

**DOI:** 10.15698/mic2019.05.676

**Published:** 2019-05-06

**Authors:** Andreas Zimmermann, Katharina Kainz, Sebastian J. Hofer, Maria A. Bauer, Sabrina Schroeder, Jörn Dengjel, Federico Pietrocola, Oliver Kepp, Christoph Ruckenstuhl, Tobias Eisenberg, Stephan J. Sigrist, Frank Madeo, Guido Kroemer, Didac Carmona-Gutierrez

**Affiliations:** 1 Institute of Molecular Biosciences, NAWI Graz, University of Graz, Graz, Austria.; 2 Division of Endocrinology and Diabetology, Department of Internal Medicine, Medical University of Graz, Graz, Austria.; 3 BioTechMed Graz, Graz, Austria.; 4 Department of Biology, Université de Fribourg, Switzerland.; 5 Institute for Research in Biomedicine; Barcelona, Spain; 6 Equipe 11 labellisée Ligue contre le Cancer, Centre de Recherche des Cordeliers, INSERM U 1138, Paris, France.; 7 Metabolomics and Cell Biology Platforms, Gustave Roussy Comprehensive Cancer Center, Villejuif, France.; 8 Université de Paris, Paris, France.; 9 Sorbonne Université Paris, France.; 10 BioHealth Graz, Graz, Austria.; 11 Central Lab Gracia, NAWI Graz, University of Graz, Graz, Austria.; 12 Institute for Biology/Genetics, Freie Universität Berlin, Berlin, Germany.; 13 Pôle de Biologie, Hôpital Européen Georges Pompidou, Paris, France.; 14 Suzhou Institute for Systems Biology, Chinese Academy of Sciences, Suzhou, China.; 15 Karolinska Institute, Department of Women's and Children's Health, Karolinska University Hospital, Stockholm, Sweden.

## Abstract

GATA transcription factors (TFs) are a conserved family of zinc-finger TFs that fulfill diverse functions across eukaryotes. Accumulating evidence suggests that GATA TFs also play a role in lifespan regulation. In a recent study, we have identified a natural compound, 4,4' dimethoxychalcone (DMC) that extends lifespan depending on reduced activity of distinct GATA TFs. Prolonged lifespan by DMC treatment depends on autophagy, a protective cellular self-cleaning mechanism. In yeast, DMC reduces the activity of the GATA TF Gln3 and, at the same time, deletion of GLN3 increases autophagy levels during cellular aging per se. Here, we examine current data on the involvement of GATA TFs in the regulation of both autophagy and lifespan in different organisms and explore, if GATA TFs are suitable targets for anti-aging interventions.

Global life expectancy at birth has increased over the past decades from 66.5 years in 2000 to 72.0 years in 2016: an increase of 5.5 years. In contrast, healthy life expectancy at birth, the number of years a person might live without disabilities, has only experienced an average increase of only 4.8 years between 2000 and 2016 1. Put simply, a person born today will likely spend the last decade of her or his life suffering from age-associated conditions, like neurodegeneration, cardiovascular disease, diabetes or cancer. Anti-aging strategies aim at closing this gap between life- and healthspan, either by behavioral - mostly dietary - interventions or by pharmacologically targeting cellular pathways that influence aging. Among these anti-aging molecules are compounds like rapamycin, which inhibits the central nutrient sensing kinase mechanistic target of rapamycin (mTOR), resveratrol, which stimulates the activity of the histone deacetylase sirtuin-1, or spermidine, which inhibits the acetyltransferase EP300 2,3,4,5. Thus far, dozens of anti-aging compounds have been described, and most of them act via decreased nutrient signaling and/or reduced protein acetylation, which seems to be a common hallmark among pharmacological anti-aging interventions 2. Nevertheless, novel molecules, especially those acting via alternative pathways, are needed, since they might be used in new combinatory approaches.

In a recent study 6, we investigated different classes of flavonoids, a group of secondary metabolites from plants, for their ability to promote longevity. For that purpose, we conducted a high-throughput screen based on chronological aging of the yeast *Saccharomyces cerevisiae*, an established model for the aging of post-mitotic cells 7,8. In particular, our screen measured three different aging-associated hallmarks as readouts: (i) the loss of plasma membrane integrity, (ii) the accumulation of reactive oxygen species, and (iii) the loss of clonogenic potential. The compound that most consistently improved all three parameters was the chalcone 4,4’-dimethoxychalcone (DMC), which we detected in the Asian traditional medicine plant *Angelika keiskei *(also known as Ashitaba). Subsequent experiments unraveled that DMC administration prolonged lifespan in nematodes (*Caenorhabditis elegans*) and fruit flies (*Drosophila melanogaster*) and decelerated cellular senescence in human cancer cells.

Many anti-aging compounds induce autophagy, an intracellular mechanism that recycles superfluous or damaged cellular material by sequestering it in vesicles. These so-called autophagosomes subsequently fuse with lysosomes, in which the cargo is degraded to replenish building blocks for anabolic reactions 2,9. DMC treatment led to elevated autophagy levels in all organisms tested, including yeast, nematodes, flies, mice and cultured human cells. DMC injection protected mice from hepatotoxicity induced by acute ethanol intoxication and from cardiovascular damage induced by prolonged ischemia, which are two conditions that can be improved by autophagy induction 6. Importantly, both lifespan extension and cardioprotection - but not liver protection - depended on intact autophagic signaling, as DMC was not able to improve these parameters upon deletion of autophagy-related (*ATG*) genes. Moreover - unlike many other anti-aging compounds - DMC treatment did not reduce mTOR signaling, and in yeast, the anti-aging effects depended neither on the mTOR component Tor1, nor on the sirtuin-1 homolog Sir2. Instead, a mechanistic screen in yeast revealed that DMC required the depletion of the GATA transcription factor (TF) Gln3 to exert its anti-aging effects. DMC treatment of wildtype cells resulted in a phenotype similar to cells devoid of Gln3: both DMC treatment and *GLN3* deletion reduced cell death and promoted autophagy during chronological aging. In addition, we observed similar changes in the abundance of amino acid in the metabolome of DMC-treated wildtype and Δ*gln3* cells. Importantly, DMC treatment reduced Gln3 signaling close to the levels of Δ*gln3* cells, altogether suggesting that DMC might mimic a Gln3-depleted status. Gln3 is a part of a zinc finger TF system, which also includes the GATA TFs Gat1, Gzf3 and Dal80. All these GATA family members recognize similar core consensus sequences and regulate nitrogen catabolite repression (NCR), which primarily serves to replenish amino acid biosynthesis when cells grow on poor nitrogen sources. GATA TFs can both activate and repress transcription of target genes, and all yeast GATA TFs are woven into a net of extensive cross-regulation and feedback loops 10. Despite the functional similarity among GATA TFs, only deletion of *GLN3*, but not of any other GATA TF, was able to mimic the anti-aging effects of DMC 6.

Gln3 translocates to the nucleus and activates NCR-responsive genes (e.g. the ammonium permease *MEP2*) upon rapamycin treatment, indicating that mTOR signaling regulates Gln3 activity 11. Since mTOR inhibition by rapamycin potently induces autophagy and Gln3 activation coincides with increased autophagic flux, it has long been suggested that Gln3 is a direct activator of genes involved in autophagy. Expression of *ATG14* is reportedly induced upon deletion of Ure2, a cytosolic protein that sequesters and inhibits both Gln3 and Gat1 12, although a more recent study has found no direct effect of* GLN3 *deletion on *ATG14* transcription 13. In chronological aging experiments, we observed that deletion of *GLN3* resulted in increased autophagic flux, and rapamycin treatment was even more effective (both when monitoring survival and autophagy) in Δ*gln3* cells, suggesting that rapamycin-induced autophagy induction and nuclear Gln3 localization are rather correlative than causal events. Importantly, Gln3 might have a different role upon acute autophagy induction, e.g. by nitrogen starvation. Indeed, ‘basal’ Gln3 activity seems to repress transcription of several ATG genes in rich media, while some *ATG* genes showed reduced transcription upon nitrogen starvation when Gln3 was absent 13. In any case, a model where basal Gln3 activity represses autophagy during chronological aging is more consistent with the reported longevity phenotype of Δ*gln3* cells 6,14. Since Gln3 regulates the expression of genes which mediate amino acid biosynthesis, it is possible that Gln3 inhibition leads to depletion of amino acids, which in turn activates autophagic flux as an alternative pathway for replenishing amino acid pools. This hypothesis is corroborated by the metabolic signature of Δ*gln3 *cells, albeit the causal mechanistic relationship between these changes and autophagy induction remains to be determined 6.

Importantly, our study corroborated that GATA TFs are involved in the beneficial effects of DMC in higher eukaryotes. Indeed, DMC failed to increase lifespan in nematodes with concomitant depletion of the Gln3 homolog elt-1. In addition, RNAi-mediated knockdown of *elt-1* led to increased autophagosome formation, and DMC did not further increase the number of autophagosomes in *elt-1* RNAi-treated animals 6. Similarly, DMC was unable to boost autophagosome formation in human cells when GATA-2 (and to a lesser extent GATA-3 and GATA-4), but not other GATA TFs were depleted.

In multicellular organisms, GATA TFs have mainly been implicated in development, particular hematopoiesis and cardiac differentiation 15,16. Interestingly, the crosstalk among different GATA TFs seems to be conserved between yeast and humans, as GATA TF switching and feedback inhibition represent important developmental control circuits in both species 17. Nevertheless, in multicellular organisms, it is poorly investigated whether GATA TFs are involved in the regulation of amino acid biosynthesis and autophagy and whether they are influenced by mTOR signaling. In hematopoietic cells, GATA-1 overexpression activates the expression of genes involved in autophagy, although some ATGs (e.g. ATG5) are repressed 18. In other tissues, GATA-4 accumulates with age and might contribute to the senescence-associated secretory phenotype and age-associated tissue inflammation, making it an attractive target for anti-aging interventions. Intriguingly, GATA-4 seems to be targeted by SQSTM1/p62 for autophagic degradation 19. It is unknown, however, if GATA-4 itself can regulate autophagy. On the other hand, in nematodes, aging is accompanied by decreasing levels of the GATA TF elt-2, and accordingly, elt-2 overexpression extends lifespan 20. In flies, the GATA TF serpent (*srp*), the closest Drosophila homolog of Gln3 has been suggested to play a role in autophagy execution, as *srp* knockdown prevents fusion of lysosomes with autophagosomes 21. In contrast, a more recent study has implicated *srp* as a negative regulator of dietary restriction-mediated lifespan extension in flies for at least two reasons. First, genes that were differentially expressed upon restriction of essential amino acids (EAA), a condition that extends longevity, were enriched for GATA TF binding sites. Second, fatbody-specific knockdown of *srp *was sufficient to increase lifespan and abolished the positive effects of EAA restriction. Of note, the signature of EAA-enriched GATA-regulated genes was also observed upon rapamycin treatment, suggesting that in Drosophila, GATA TF signaling might be regulated by mTOR. Interestingly, whole-body *srp* knockdown had a similar effect on lifespan as dietary restriction, but reduced fecundity, suggesting that systemic depletion of this TF comes at a cost 22. Our data yielded a similar picture: systemic elt-1 depletion in nematodes failed to increase lifespan, yet abolished the anti-aging effect of DMC 6. As a possibility, in multicellular organisms, tissue-specific depletion of GATA TFs might yield life- or healthspan effects that cannot be seen upon whole-body depletion, but this needs to be further investigated.

Apparently, longevity-extending effects of DMC rely on an increase in autophagic flux. Still, several questions regarding the mode of action of DMC remain to be answered:

(i) how does DMC inhibit Gln3? We could not demonstrate any physical interaction of the compound with the Gln3 protein. In fact, DMC might target Gln3 indirectly by acting on Gln3 regulators. Besides mTOR-mediated regulation, Gln3 activity can be modulated by PP2A-like protein phosphatases, although in many cases, mTOR activity inversely correlates with PP2A activity 23. Intriguingly, DMC-mediated cytoprotection was lost in yeast strains lacking the PP2A subunits Tpd3 or Pph21/Pph22 6. Possible direct effects of DMC on such PP2A-like enzymes should be studied in the future.

(ii) Does DMC reduce GATA signaling in multicellular organisms? While we did not measure GATA activity in multicellular models, two pieces of information suggest that this could be the case. First, we found metabolomic lterations in the liver and heart tissues from DMC-treated mice, particularly in amino acid-related metabolism, that are reminiscent of those found in yeast. Second, as in yeast, the pro-autophagic DMC effects are dependent on GATA TFs in nematodes and human cell culture. However, it remains to be formally demonstrated that these changes correlate with reduced GATA signaling.

(iii) Are there other factors involved in the pro-autophagic activity of DMC? Although our data indicate that DMC promotes autophagy in an mTOR-independent manner and requires GATA signaling, we cannot exclude further mechanistic determinants. For instance, the activity of other TFs might be required for, or influenced by GATA TFs in the context of aging. The likely complexity of such a GATA-connected transcriptional network will be a matter of further investigation. In fact, in mammals, the transcriptional regulation of autophagy alone encompasses a set of at least 20 TFs, including FOXO1/3, ATF4 and TFEB 24. So far, except for GATA-1, GATA TFs have not been part of the transcriptional landscape of autophagy regulation. Again, future studies will have to explore to what extent GATA TFs are involved in the transcriptional control of autophagy, likely in a cell type-dependent fashion given the tissue-specific expression profile of GATA TFs in mammals 25 and the dependency of caloric restriction on GATA signaling in Drosophila 22.

GATA TF signaling has diverse functions across different phyla. It is not unlikely that an ancient system of interdependent paralogous TFs has evolved to fulfil different needs in distinc species, ranging from tight regulation of nitrogen sensing in yeast to developmental switches in multicellular organisms. Nonetheless, emerging evidence suggests that more regulatory functions are conserved across species than previously expected, although tissue-specific effects of GATA TF modulation have to be taken into account. Arguably, this complicates pharmacological anti-aging interventions, as compounds will either have to modulate GATA TF activity in a localized manner or specifically target GATA TF subtypes. Despite these constraints, accumulating data place GATA TFs in the limelight as actionable targets for postponing age-associated disease.

## References

[1] WHO. GHO | By category | Life expectancy and Healthy life expectancy - Data by WHO region.. http://apps.who.int/gho/data/view.main.SDG2016LEXREGv?lang=en.

[2] Madeo F, Carmona-Gutierrez D, Hofer SJ, Kroemer G (2019). Caloric Restriction Mimetics against Age-Associated Disease: Targets, Mechanisms, and Therapeutic Potential.. Cell Metab.

[3] Pietrocola F, Lachkar S, Enot DP, Niso-Santano M, Bravo-San Pedro JM, Sica V, Izzo V, Maiuri MC, Madeo F, Mariño G, Kroemer G (2015). Spermidine induces autophagy by inhibiting the acetyltransferase EP300.. Cell Death Differ.

[4] Eisenberg T (2016). Cardioprotection and lifespan extension by the natural polyamine spermidine.. Nat Med.

[5] Eisenberg T, Knauer H, Schauer A, Büttner S, Ruckenstuhl C, Carmona-Gutierrez D, Ring J, Schroeder S, Magnes C, Antonacci L, Fussi H, Deszcz L, Hartl R, Schraml E, Criollo A, Megalou E, Weiskopf D, Laun P, Heeren G, Breitenbach M, Grubeck-Loebenstein B, Herker E, Fahrenkrog B, Fröhlich K-U, Sinner F, Tavernarakis N, Minois N, Kroemer G, Madeo F (2009). Induction of autophagy by spermidine promotes longevity.. Nat Cell Biol.

[6] Carmona-Gutierrez D (2019). The flavonoid 4,4′-dimethoxychalcone promotes autophagy-dependent longevity across species.. Nat Commun.

[7] Longo VD, Fabrizio P (2012). Chronological Aging in Saccharomyces cerevisiae.. Subcell Biochem.

[8] Zimmermann A, Hofer S, Pendl T, Kainz K, Madeo F, Carmona-Gutierrez D (2018). Yeast as a tool to identify anti-aging compounds.. FEMS Yeast Res.

[9] Yin Z, Pascual C, Klionsky DJ (2016). Autophagy: machinery and regulation.. Microbial Cell.

[10] Ljungdahl PO, Daignan-Fornier B (2012). Regulation of Amino Acid, Nucleotide, and Phosphate Metabolism in Saccharomyces cerevisiae.. Genetics.

[11] Tate JJ, Georis I, Feller A, Dubois E, Cooper TG (2009). Rapamycin-induced Gln3 Dephosphorylation Is Insufficient for Nuclear Localization: Sit4 AND PP2A PHOSPHATASES ARE REGULATED AND FUNCTION DIFFERENTLY.. J Biol Chem.

[12] Chan T-F, Bertram PG, Ai W, Zheng XFS (2001). Regulation of APG14 Expression by the GATA-type Transcription Factor Gln3p.. J Biol Chem.

[13] Bernard A, Jin M, Xu Z, Klionsky DJ (2015). A large-scale analysis of autophagy-related gene expression identifies new regulators of autophagy.. Autophagy.

[14] Powers RW (2006). Extension of chronological life span in yeast by decreased TOR pathway signaling.. Genes Dev.

[15] Cohen ML, Kim S, Morita K, Kim SH, Han M (2015). The GATA Factor elt-1 Regulates C.. elegans Developmental Timing by Promoting Expression of the let-7 Family MicroRNAs. PLOS Genet.

[16] Tremblay M, Sanchez-Ferras O, Bouchard M (2018). GATA transcription factors in development and disease.. Development.

[17] Bresnick EH, Lee H-Y, Fujiwara T, Johnson KD, Keles S (2010). GATA Switches as Developmental Drivers.. J Biol Chem.

[18] Kang Y-A, Sanalkumar R, O’Geen H, Linnemann AK, Chang C-J, Bouhassira EE, Farnham PJ, Keles S, Bresnick EH (2012). Autophagy Driven by a Master Regulator of Hematopoiesis.. Mol Cell Biol.

[19] Kang C, Xu Q, Martin TD, Li MZ, Demaria M, Aron L, Lu T, Yankner BA, Campisi J, Elledge SJ (2015). The DNA damage response induces inflammation and senescence by inhibiting autophagy of GATA4.. Science.

[20] Mann FG, Van Nostrand EL, Friedland AE, Liu X, Kim SK (2016). Deactivation of the GATA Transcription Factor ELT-2 Is a Major Driver of Normal Aging in C.. elegans. PLOS Genet.

[21] Bánréti Á, Lukácsovich T, Csikós G, Erdélyi M, Sass M (2012). PP2A regulates autophagy in two alternative ways in Drosophila.. Autophagy.

[22] Dobson AJ, He X, Blanc E, Bolukbasi E, Feseha Y, Yang M, Piper MDW (2018). Tissue-specific transcriptome profiling of Drosophila reveals roles for GATA transcription factors in longevity by dietary restriction.. Npj Aging Mech Dis.

[23] Tate JJ, Georis I, Dubois E, Cooper TG (2010). Distinct Phosphatase Requirements and GATA Factor Responses to Nitrogen Catabolite Repression and Rapamycin Treatment in Saccharomyces cerevisiae.. J Biol Chem.

[24] Feng Y, Yao Z, Klionsky DJ (2015). How to control self-digestion: transcriptional, post-transcriptional, and post-translational regulation of autophagy.. Trends Cell Biol.

[25] Lentjes MH, Niessen HE, Akiyama Y, de Bruïne AP, Melotte V, van Engeland M (2016). The emerging role of GATA transcription factors in development and disease.. Expert Rev Mol Med.

